# Mass spectral determination of phosphopantetheinylation specificity for carrier proteins in *Mycobacterium tuberculosis*

**DOI:** 10.1002/2211-5463.12140

**Published:** 2016-10-24

**Authors:** James Jung, Ghader Bashiri, Jodie M. Johnston, Edward N. Baker

**Affiliations:** ^1^ Maurice Wilkins Centre for Molecular Biodiscovery and School of Biological SciencesThe University of AucklandNew Zealand; ^2^ Present address: W. M. Keck Structural Biology LaboratoryCold Spring Harbor Laboratory1 Bungtown RoadCold Spring HarborNY11724USA

**Keywords:** carrier proteins, mass spectrometry, mutagenesis, *Mycobacterium tuberculosis*, phosphopantetheinyl transferases

## Abstract

Phosphopantetheinyl transferases (PPTases) are key elements in the modular syntheses performed by multienzyme systems such as polyketide synthases. PPTases transfer phosphopantetheine derivatives from Coenzyme A to carrier proteins (CPs), thus orchestrating substrate supply. We describe an efficient mass spectrometry‐based protocol for determining CP specificity for a particular PPTase in organisms possessing several candidate PPTases. We show that the CPs MbtL and PpsC, both involved in synthesis of essential metabolites in *Mycobacterium tuberculosis*, are exclusively activated by the type 2 PPTase PptT and not the type 1 AcpS. The assay also enables conclusive identification of the reactive serine on each CP.

**Abbreviations**
4′‐PP: phosphopantetheine
ACP: acyl carrier protein
ArCP: aryl carrier protein
CP: carrier protein
EMSA: electrophoretic mobility shift assay
FAS: fatty acid synthase
IMAC: immobilised metal affinity chromatography
LC‐MS: liquid chromatography mass spectrometry
*Mtb*: *Mycobacterium tuberculosis*
NRPS: non‐ribosomal peptide synthase
PCP: peptidyl carrier protein
PDIM: phthiocerol dimycocerosate
PKS: polyketide synthase
PPTase: phosphopantetheinyl transferase
SEC: size‐exclusion chromatography
TB: tuberculosis
TCEP: tris(2‐carboxyethyl)phosphine
WT: wild‐type

Phosphopantetheinylation is a post‐translational modification that is essential across all three domains of life 1, 2. Phosphopantetheine‐dependent biosynthetic pathways resemble modular production lines 3. Carrier protein (CP) modules act as molecular conveyor belts carrying the metabolic intermediates, covalently tethered to the long and flexible phosphopantetheine (4′‐PP) arm, from one reaction centre to the next. 4′‐PP attachment to CPs is thus essential for the activity of key biosynthetic pathways and ultimately for the viability of the organisms. CPs are present as acyl carrier proteins (ACPs) in fatty acid synthase (FAS) and polyketide synthase (PKS) systems and as peptidyl carrier proteins (PCPs) and aryl carrier proteins (ArCPs) in nonribosomal peptide synthase (NRPS) systems 2.

Phosphopantetheinyl transferases (PPTases) play a crucial role in this process, binding CoA and transferring its 4′‐PP moiety to a conserved Ser residue on CPs. This converts inactive apo‐CPs to their functional holo‐forms 2. Two common types of PPTase can be found in various organisms, classified on the basis of their structural organisation. Type‐I PPTases are homotrimers and are generally thought to activate ACPs of FASs carrying out primary lipid metabolism 1, 4, 5. On the other hand, type‐II PPTases are monomers and are generally thought to activate CPs of PKSs and NRPSs involved in secondary metabolism.

*Mycobacterium tuberculosis* (*Mtb*), the causative agent of Tuberculosis (TB), possesses both types of PPTase, AcpS (type‐I) 4, 6 and PptT (type‐II) 7, 8, which are together assumed to be responsible for activating the more than 20 different CPs encoded in the *Mtb* genome 9, 10. These target CPs have crucial roles in the biology and pathogenesis of *Mtb*, suggesting that the PPTases that activate them could be useful targets for the design of anti‐TB drugs. In most cases, however, it is not known which PPTase is responsible for activating a particular CP. The importance of direct experimental determination of the correct PPTase has been shown for AcpM (Rv2244), a discrete ACP protein central to *Mtb* FAS‐II, which provides lipid precursors for various secondary metabolites, including mycolic acids. AcpM can be activated by the *Escherichia coli* type‐I AcpS when expressed in that organism, but has been shown to be activated in *Mtb* by the type‐II PPTase PptT 11.

Here, we have examined the activation of CPs involved in the biosynthesis of two secondary metabolites critical to *Mtb* biology. MbtL (Rv1344) is an ACP protein that carries lipid moieties destined for the mycobacterial siderophores mycobactin (membrane‐associated) and carboxymycobactin (extracellular) 12, 13, 14, 15, 16, 17, 18, 19. The *Mtb*‐PPTase responsible for activating MbtL has not been determined, although *B. subtilis* Sfp has previously been used as a surrogate to phosphopantetheinylate MbtL 15. PpsC (Rv2933) is a PKS that mediates the biosynthesis of the mycobacterial polyketide lipid virulence factors known as phthiocerol dimycocerosates (PDIMs) 20. There is one ACP domain (residues 2042–2188) within PpsC 21, 22, which has been shown by electrophoretic mobility shift assay (EMSA) to be activated by PptT, but has not been tested against AcpS 21.

In this report we have used a straightforward and definitive mass spectrometry‐based protocol for determining substrate CP specificities, applying it to the two PPTases from *Mtb*, AcpS and PptT. We show that PptT is the sole PPTase responsible for activating MbtL in mycobactin biosynthesis, and that AcpS cannot activate PpsC, which is thus fully specific for PptT. This analysis also enables us to confirm the proposed 4′‐PP attachment sites of these CPs.

## Materials and methods

### Cloning and mutagenesis

*Mtb*‐PptT was cloned, expressed and purified as an MBP‐fusion construct using a previously reported protocol 8. The ORFs encoding *acpS*,* mbtL* and *ppsC‐ACP* were amplified by PCR from *M. tuberculosis* H37Rv genomic DNA using PrimeSTAR HS DNA polymerase (Takara Bio, Mountain View, CA, USA) and primers listed in Table 1. The ORFs were then cloned into the pYUBDuet shuttle vector 23, 24 using *Bam*HI and *Hin*dIII restriction sites, expressing the proteins as N‐terminal His_6_‐tagged constructs. Mutant constructs with the putative recipient Ser mutated into a nonreactive Ala residue (MbtL S63A and PpsC S2106A) were created from the wild‐type (WT) constructs by site‐directed mutagenesis using *Pfu*Ultra II DNA polymerase (Agilent Technologies, Santa Clara, CA, USA) and primers listed in Table 1.

**Table 1:** Primer sequences used for cloning and mutagenesis. The introduced point mutations in the sequences are coloured in red

Constructs	Primer sequences (5′–3′)
WT‐PpsC‐ACP	Forward: CTACTTGGATCCGCATGACTCGGCGGCCCGCAAAAG
	Reverse: CTACTTAAGCTTTCATGACTCGCCTCGCGTCGCAG
PpsC‐ACP S2106A	Forward: GGACTCGACGCGCTGATGGGC
	Reverse: CAGGGTTTCCAGCGGTCGGTGG
WT‐MbtL	Forward: CTACTTGGATCCGATGTGGCGATATCCACTAAGTACAAGGCTAG
	Reverse: CTACTTAAGCTTTCACTCATCGCGGTATTTGGCCGCG
MbtL S63A	Forward: TGGGACTGGATGCGGTGGCCTTC
	Reverse: CATCGTCGACCAACCTGGCATCAGG

### Protein expression and purification

Expression of *Mtb*‐AcpS, MbtL and PpsC‐ACP constructs was carried out using *E. coli* C41 (DE3) cells with autoinduction protocols 25; cells were grown at 37 °C for 4 h, then at 18 °C overnight. All media were supplemented with 50 μg·mL^−1^ hygromycin B. Cells were lysed using a cell disruptor (Microfluidics, Westwood, MA, USA) at 18 500 psi in 20 mm Tris‐HCl pH 7.5, 300 mm NaCl, 10% (v/v) glycerol, 0.5 mm tris(2‐carboxyethyl)phosphine (TCEP) and 30 mm imidazole. After centrifugation, the His_6_‐tagged proteins were purified from the supernatant using a Ni‐nitrilotriacetic acid‐immobilised metal affinity chromatography (IMAC) column (Macherey‐Nagel, Duren, Germany). After washing the column with the lysis buffer, the bound proteins were eluted with a linear imidazole gradient (30–500 mm). Further purification utilised size‐exclusion chromatography (HiLoad 10/300 Superdex 200; GE Healthcare, Chicago, IL, USA) in buffer without imidazole. Elution fractions containing CP proteins were pooled and concentrated. The *Mtb*‐AcpS and *Mtb*‐PptT constructs were both shown to possess phosphopantetheinyl transferase activity *in vitro* using a previously reported assay 26.

### Phosphopantetheinylation assay and mass spectrometry

Each type of WT‐CP (30 nm) was mixed with either *Mtb*‐AcpS (5 μm) or *Mtb*‐PptT (5 μm) together with CoA (0.5 mm) and MgCl_2_ (1 mm) and made up to 100 μL with the SEC buffer. The reaction was carried out at 30 °C for 2 h and the resulting samples were then analysed by electrospray ionisation liquid chromatography mass spectrometry (LC‐MS) using a QSTAR XL Hybrid LC‐MS/MS spectrometer (Applied Biosystems, Auckland, New Zealand). The samples were separated on a C5 or C8 reverse‐phase high‐pressure liquid chromatography column, looking for a mass addition consistent with attachment of phosphopantetheine (C_11_H_21_N_2_O_6_PS, 340.3 Da) to a WT apo‐CP. The same experiments were also set up with the nonreactive Ser→Ala mutant CPs as negative control experiments. Further negative experiments were set up with WT‐CP and no PPTase added.

## Results and Discussion

### MbtL is activated by PptT

**Figure 1:**
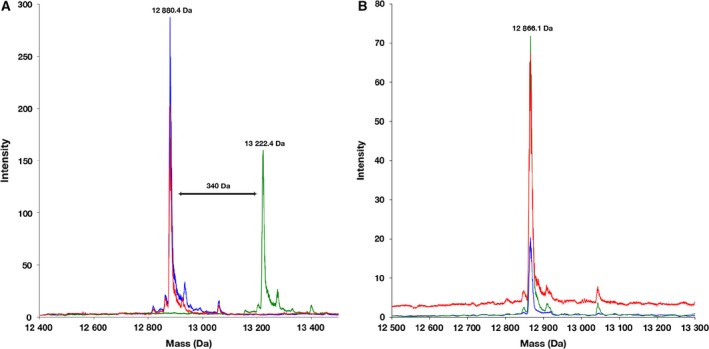
Mass spectra of MbtL activation by PptT. (A) An overlay of deconvoluted mass spectra, showing WT‐MbtL without PPTases added (blue), WT‐MbtL + AcpS (red) and WT‐MbtL + PptT (green). The positive mass shift in MbtL (from 12 880.4 Da to 13 222.4 Da) when mixed with PptT is consistent with attachment of a phosphopantetheine group (340.3 Da). (B) An overlay of deconvoluted mass spectra, showing nonreactive Ser63Ala mutant‐MbtL without PPTases added (blue), mutant‐MbtL + AcpS (red) and mutant‐MbtL + PptT (green). The mass of mutant MbtL (12 866.1 Da) is consistent with the calculated mass value (12 996.6 Da) with the N‐terminal Met excised and is unchanged when mixed with AcpS or PptT. The intensity values are in counts per second.

MbtL is an ACP involved in mycobacterial siderophore biosynthesis, mediating fatty acid substitutions on the lysine moiety of mycobactins 12, 13, 14, 15, 16, 17, 18, 19. We tested whether it is activated by PptT rather than AcpS, as might be supposed from its role in secondary metabolism. Using LC‐MS, a peak at 12 880.4 Da was observed for WT MbtL samples (Fig. 1A), consistent with the calculated MW (13 012.6 Da) minus the N‐terminal Met (−131.2 Da) 27. A new peak at 13 222.4 Da, corresponding to a phosphopantetheine adduct (340.3 Da), appeared when MbtL was incubated with PptT but not when it was incubated with AcpS (Fig. 1A). No mass addition was observed in the negative control reaction in which no PPTase was added (Fig. 1A, red trace). PptT is therefore identified as the PPTase that activates MbtL in *Mtb*.

The same assay can be used to confirm Ser63 as the 4′‐PP attachment site of MbtL, as no mass addition was observed in reactions using the Ser63Ala mutant MbtL (Fig. 1B). Identification of the reactive serine residue in CPs is nontrivial, as although it is usually located within a short signature sequence motif (D/H)S(L/I) 28, 29, variations do occur. In the case of MbtL, we noted that Ser63 was the only serine to be followed by an aliphatic hydrophobic residue similar to Leu/Ile. The confirmation that Ser63 is the reactive serine expands the consensus sequence motif to (D/H)S(L/I/V), which may be useful for identifying new CPs and their activation sites in the future.

### PpsC‐ACP is activated by PptT but not by AcpS

**Figure 2:**
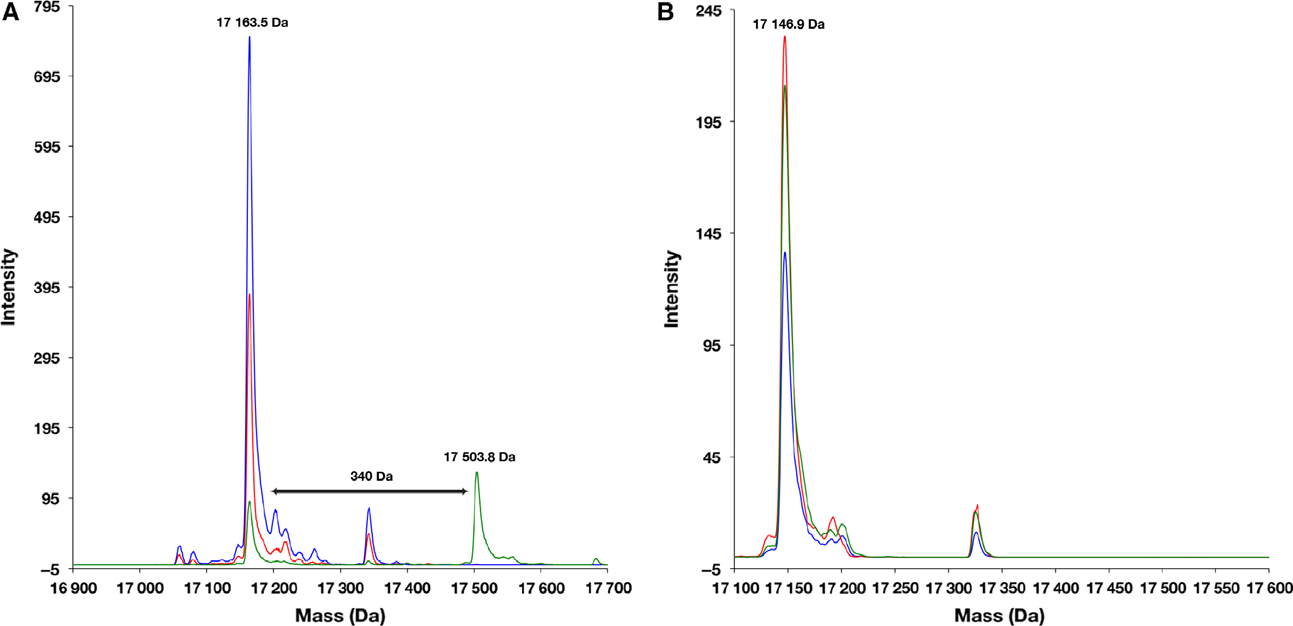
Mass spectra of PpsC activation by PptT. (A) An overlay of deconvoluted mass spectra, showing WT‐PpsC without PPTases added (blue), WT‐PpsC + AcpS (red) and WT‐PpsC + PptT (green). The positive shift in PpsC mass (from 17 163.5 Da to 17 503.8 Da) when mixed with PptT is consistent with the attachment of a phosphopantetheine group (340.3 Da). (B) An overlay of deconvoluted mass spectra, showing nonreactive Ser2106Ala mutant PpsC‐ACP without PPTases added (blue), mutant PpsC‐ACP + AcpS (red) and mutant PpsC‐ACP + PptT (green). The mass of mutant PpsC‐ACP (17 146.7 Da) is consistent with the calculated mass value (17 279.1 Da) with the N‐terminal Met excised and is unchanged when mixed with AcpS or PptT.

PpsC‐ACP mediates the biosynthesis of mycobacterial polyketide lipid virulence factors. We tested PpsC‐ACP for reactivity with both PptT and AcpS, since although it has been found to be activated by PptT 21, no tests have been reported against AcpS. A peak at 17 163.5 Da was observed in the WT‐PpsC‐ACP samples (Fig. 2A), consistent with the calculated MW (17 295.1 Da) without the N‐terminal Met residue. A new peak at 17 503.8 Da, corresponding to a phosphopantetheine adduct (340.3 Da), appeared in the reaction with PptT, but not with AcpS, confirming that PptT is the sole activator of PpsC‐ACP. No mass addition was observed in the negative control reactions without PPTases added. Mutation of Ser2106 to a nonreactive Ala residue confirmed this residue as the 4′‐PP attachment site of PpsC‐ACP, as no mass addition was observed in reactions using the Ser2106Ala mutant construct (Fig. 2B).

### CP activation in *M. tuberculosis*

The two PPTases in *Mtb*, AcpS and PptT, have each been shown to be independently essential, pointing to their importance for activating the CPs from key biosynthetic processes. There is thus great interest in both the *Mtb* CPs and their associated PPTases as potential anti‐TB drug targets 9, 10, 30. There are over 20 different CPs in *Mtb* that are potential substrates for activation by AcpS and PptT 9. Determination of the correct *Mtb* PPTase‐CP pairing is important to extend our understanding of the physiological roles played by the two PPTases, and to predict the likely outcomes of developing inhibitors against them. Activation of a CP by more than one specific PPTase could require coinhibition of all activating PPTases as drug targets.

Our MS‐based PPTase assay, in which we incubated CPs with both of the *Mtb* PPTases, enables the unequivocal determination of the PPTase responsible for activating each CP and also enables determination of the 4′‐PP attachment site within each CP. The demonstration that both MbtL, involved in the biosynthesis of mycobactin siderophores, and the ACP domain of PpsC, which mediates the biosynthesis of the mycobacterial polyketide lipid virulence factors known as phthiocerol dimycocerosates (PDIMs), are exclusively activated by PptT and not by AcpS, underscores the individual roles of these two PPTases. Taken together with the activation of AcpM exclusively by PptT in *Mtb* 11, this supports the view that class II PPTases such as PptT tend to be preferentially used in secondary metabolism. Nevertheless, the fact that in a different biological environment (expression in *E. coli*) AcpM can also be activated by the class I *E. coli* AcpS emphasises the importance of definitive experimental determination of the relevant PPTase for any CP activation.

## Conclusions

The MS‐based functional assay used here, similar to that used for analysis of the activation of AcpM 11, provides a simple and definitive experimental method for identification of the particular PPTases involved in activation of any given CP. This is of particular importance in organisms that possess more than one PPTase, such as *Mtb*. The putative reactive serine in a CP can also be definitively identified by using a Ser→Ala mutant of the CP in the same assay. Using this protocol we have shown that two CPs in *Mtb*, MbtL of mycobactin biosynthesis and PpsC of PDIM biosynthesis, are exclusively activated by the class II PPTase PptT, and not by the class I AcpS. Coupled with the fact that two other CPs of mycobactin biosynthesis, MbtB and MbtE, are also reported to be activated by PptT 31, this is consistent with the proposal that in organisms with both types of PPTase the type‐II transferases tend to be specific for CPs of secondary metabolism, whereas type‐I are specific for the ACP of FAS. Similar proposals have also been made for *Vibrio cholerae* 32, 33, and for *Staphylococcus aureus* 32, 34, each of which has both a type‐I and a type‐II PPTase.

## Author contributions

ENB and JJ initiated the study. JJ carried out the experimented work and collected and analysed the data. GB and JMJ were advisors on the experimental work. JJ and ENB wrote the manuscript, with help from GB and JMJ.
